# Renal medullary carcinoma: a systematic review of clinical and radiological findings

**DOI:** 10.1007/s11255-026-05113-4

**Published:** 2026-04-09

**Authors:** William Weston, Cherry Sit, Derfel Ap Dafydd, Christian Kelly-Morland, Aslam Sohaib, Shraddha Adamane, Thubeena Manickavasagar, Lisa Pickering, David Nicol, Samuel J. Withey

**Affiliations:** 1 Department of Radiology, The Royal Marsden NHS Foundation Trust, London, UK; 2 Department of Radiology, Guy’s and St Thomas’ NHS Foundation Trust, London, UK; 3 Department of Pathology, The Royal Marsden NHS Foundation Trust, London, UK; 4 Department of Medical Oncology, The Royal Marsden NHS Foundation Trust, London, UK; 5 Department of Urology, The Royal Marsden NHS Foundation Trust, London, UK; 6 Research Institute for Medical Innovation, RadboudUMC, Nijmegen, Netherlands

**Keywords:** Renal medullary carcinoma, Renal medullary neoplasm, Sickle cell haemoglobinopathy, Renal cell carcinoma

## Abstract

**Background and aims:**

Renal medullary carcinoma (RMC) is a rare subtype of renal cell carcinoma, typically described in young patients with sickle cell trait, which follows an aggressive course. We aimed to perform a systematic review of all published cases of RMC from 1995 to 2024, to summarise the clinico-pathological and radiological features.

**Methods:**

A systematic review of all published cases of RMC from 1995 to 2024 was performed. Patient demographics, clinical presentation, radiological features, metastatic profile, management, and overall survival were recorded. Sub-analysis was performed, grouped by sickle cell status.

**Results:**

219 cases from 103 articles were included, in addition to 5 previously unreported cases from our institution. 88% of patients had sickle cell haemoglobinopathy (SCH) (*n* = 168/192), with presentation at a younger age in patients with SCH (27.3 ± 13.7 versus 34.1 ± 19.1 years, *p* = 0.001). RMC was more frequently observed in the right kidney (64.6%, *n* = 122/189) than the left kidney (35.4%, *n* = 67/189) (*p* = < 0.001). Radiologically, the most common features were a central/endophytic location, hypo-enhancement relative to the adjacent cortex, and infiltrative margins. 20.7% (*n* = 42/203) were stage III at presentation and 70.9% (*n* = 144/203) of patients were Stage IV. Overall survival at 3 years was 74% for Stage I–II disease, 23% for Stage III disease, and 9% for Stage IV disease.

**Conclusions:**

Regardless of sickle cell status, RMC was shown to present at a relatively young age. It was classically seen as an infiltrative, poorly defined, endophytic tumour, centred on the renal medulla. RMC was found to be more commonly advanced at presentation and the overall survival in patients with nodal or distant metastases was poor.

**Supplementary Information:**

The online version contains supplementary material available at 10.1007/s11255-026-05113-4.

## Introduction

Renal medullary carcinoma (RMC) was first described in 1995 by Davis et al., seen as an aggressive renal cancer predominating in young patients with sickle cell trait (SCT) [1]. The prognosis in the original series was dire, with a median time from development of symptoms to death of 21 weeks [1]. Over the past 25 years, many more cases have been described, but it still represents less than 0.5% of all renal cell carcinomas (RCC) [2]. RMC has been known as SMARCB1-deficient RCC since the WHO 2022 update, and is still predominantly seen in patients with sickle cell haemoglobinopathy (SCH) [3]. A cohort of patients *without* SCH has been identified, grouped as either RCC unclassified with medullary phenotype, or SMARCB1-deficient renal RCC (without haemoglobinopathy) [3].

Despite advances in diagnostic techniques and treatment options, RMC is still diagnosed at a late stage and behaves aggressively with early metastatic disease spread. Understanding the radiological features of RMC can raise suspicion and potentially hasten diagnosis and management. Therefore, we aimed to perform a systematic review of the literature to identify cases of RMC, to summarise the patient demographics, presenting symptoms, radiological features, metastatic profile, management, and overall survival (OS).

## Methods

### Data sources and search strategy

A PubMed/MEDLINE database search was performed to identify all case reports, case series, and database studies of RMC. Article titles and abstracts were searched for “Renal” AND “Medullary” AND “Carcinoma”, combined using Boolean logic. Only articles published since 1995 were included, as this was when RMC was first described by Davis et al. [1].

Results were limited to human studies, and only those in English, Spanish, or French were included. Relevant review article reference lists were read in full to ensure appropriate studies had not been missed. The search was performed independently by three readers (SJW, CS & WW) with any disagreements resolved by consensus. In addition, five previously unpublished cases from the authors’ institution were included.

### Selection criteria

For those abstracts describing renal medullary carcinoma, the full articles were retrieved, and the following exclusion criteria were applied: already included cases or insufficient description of features (demographic, tumour characteristics, clinical presentation, imaging, management, or survival outcomes). An excluded study log recorded the reasons for exclusion.

### Data extraction

Data were extracted from the included full text articles and recorded in a database (Excel, Microsoft, Redmond WA, USA). For each article the publication details (first author, year, and country of origin), patient demographics (age, gender, race, sickle cell status), clinical features (presenting symptoms, stage at time of diagnosis, sites of metastatic disease at diagnosis, management, metastases documented at any point, OS), and imaging features (site, size, and presence or absence of tumour features) were recorded where available.

### Statistical methods

All statistical analyses were performed using RStudio (R version 4.4.2) for statistical comparisons. The independent *T*-test and Chi-square test were used for comparing patient characteristics (for age and size, an unpaired *T*-test was used; for gender, laterality, and stage at presentation, a Chi-square test was used). Kaplan–Meier plots were used to represent OS, with Chi-square test used to assess for differences in OS between stages at presentation. All tests were two-sided, and *p* < 0.05 was considered statistically significant. OS was estimated using the Kaplan–Meier method. Two-sided 95% confidence intervals were calculated using Greenwood’s variance with log (− log) transformation. Survival estimates at 12, 24, and 36 months are reported with numbers at risk at each landmark.

## Results

**Fig. 1:**
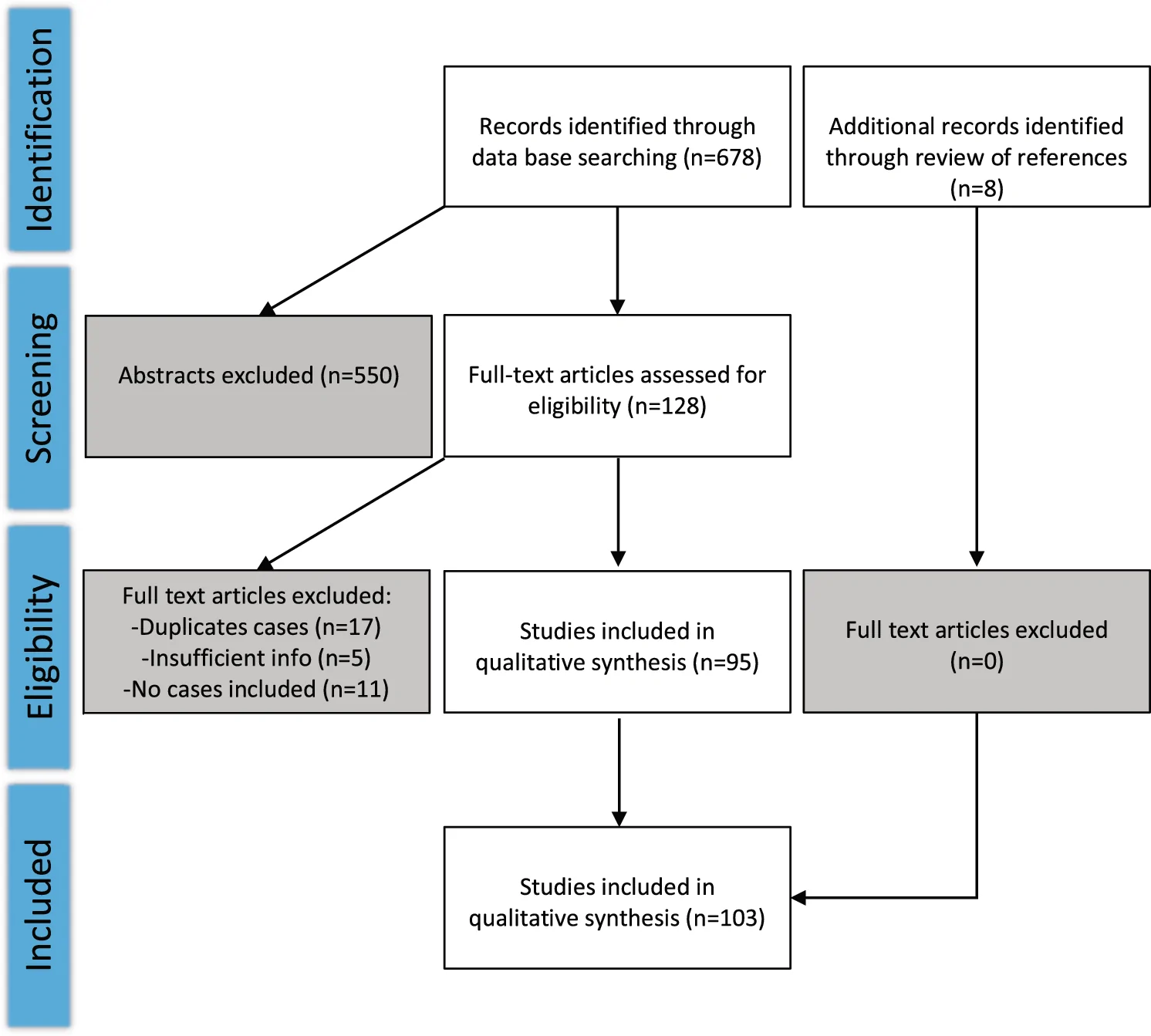
PRISMA (Preferred Reporting Items for Systematic Reviews and Meta-Analyses) guidelines for transparent reporting of systematic reviews: flowchart of article identification

PRISMA (Preferred Reporting Items for Systematic Reviews and Meta-Analyses) guidelines for the transparent reporting of systematic reviews were followed (Fig. 1).

### Eligible studies

The initial search performed on 11th November 2024 yielded 678 results. 550 articles were excluded following evaluation of their abstracts. The remaining 128 articles were retrieved in full text, with 95 studies containing sufficient information to be included in the review. An additional 8 articles were added following review of references. The full list of included articles is available in the Supplementary Material.

Of the 103 articles included, the majority originated from the USA (*n* = 71). Articles were also from: the UK (*n* = 5), Italy and France (*n* = 4 each); Japan and China (*n* = 3 each); Brazil and Canada (*n* = 2 each); and Colombia, India, Jordan, Mexico, Nigeria, Puerto Rico, Spain, Tunisia and Turkey (*n* = 1 each).

Articles from all years since 1995 were included, with a mean of 3.4 articles per year, peaking at 8 in 2017. The mean number of included cases per article was 2. 76 articles included only a single case. One article included 36 patients with usable data [4].

In total, 219 patients were included from journal articles, with an additional five previously unreported cases from our institution.

### Patient demographics

Patient demographics are described in Table 1. Information on ethnicity was available in 80.8% (*n* = 181/224); of these, 134 patients were Black (74.0%), 25 Caucasian (13.8%), 14 Asian (7.7%), 6 Hispanic (3.3%), and 2 mixed race (1.1%).

**Table 1:** Characteristics of patients with medullary renal carcinoma, grouped by sickle cell status

	Total population	Sickle cell haemoglobinopathy	Non-sickle	*P*-value
Age ± SD/years	27.5 (± 14.1) years (*n* = 188)	27.3 (± 13.7) years (*n* = 168)	34.1 (± 19.1) years (*n* = 20)	< 0.001
*Gender*				
Male	72.4% (*n* = 160/221)	77.4% (*n* = 130/168)	71.4% (*n* = 20/28)	0.92
Female	27.6% (*n* = 61/221)	22.6% (*n* = 38/168)	28.6% (*n* = 8/28)	
*Laterality*				
Right	64.6% (*n* = 122/189)	71.1% (*n* = 96/135)	59.3% (*n* = 16/27)	0.32
Left	35.4% (*n* = 67/189)	28.9% (*n* = 39/135)	40.7% (*n* = 11/27)	
Size/cm (± SD)	6.4 cm ± 2.9 cm (*n* = 127)	6.5 cm ± 2.9 cm (*n* = 95)	6.3 cm ± 3.9 cm (*n* = 24)	0.46
*Ethnicity (n* = *181)*				
Black	74.0% (*n* = 134)			
Caucasian	13.8% (*n* = 25)			
Asian	7.7% (*n* = 14)			
Hispanic	3.3% (*n* = 6)			
Mixed	1.1% (*n* = 2)			
*Presenting symptoms (n* = *183)*				
Haematuria	59.6% (*n* = 109)			
Abdominal pain	54.1% (*n* = 99)			
Palpable mass	6.0% (*n* = 11)			
Back pain	10.4% (*n* = 19)			
Systemic symptoms	24.0% (*n* = 44)			
Incidental	2.7% (*n* = 5)			
Imaging modalities in initial workup (*n* = 143)				
CT	98.6% (*n* = 141)			
US	24.5% (*n* = 35)			
MRI	10.5% (*n* = 15)			
18F-FDG PET/CT	6.3% (*n* = 9)			
Excretory urography	7.7% (*n* = 11)			
*Stage at presentation*				
I—II	8.4% (*n* = 17/203)	7.0% (*n* = 11/157)	14.3% (*n* = 4/28)	0.007
III	20.7% (*n* = 42/203)	17.8% (*n* = 28/157)	35.7% (*n* = 10/28)	
IV	70.9% (*n* = 144/203)	75.2% (*n* = 118/157)	50.0% (*n* = 14/28)	
*Treatment (n* = *183)*				
Nephrectomy	71.0% (*n* = 130)			
Metastasectomy	9.3% (*n* = 17)			
Chemotherapy	59.6% (*n* = 109)			
Immunotherapy	18.0% (*n* = 33)			
Radiotherapy	8.7% (*n* = 16)			
Palliative care only	6.6% (*n* = 12)			

Sickle cell status was known for 85.7% of patients (*n* = 192/224). Of these, 168 had SCH (87.5%), including 144 with SCT, 8 with SCD, and a further 16 patients with a suggested new diagnosis of either sickle SCT or SCD, based upon the presence of sickled erythrocytes in the nephrectomy specimens. There were additional patients with *HbSC*, *beta thalassaemia*, and one patient with *HbS, beta thalassaemia and concomitant alpha thalassaemia*.

**Fig. 2:**
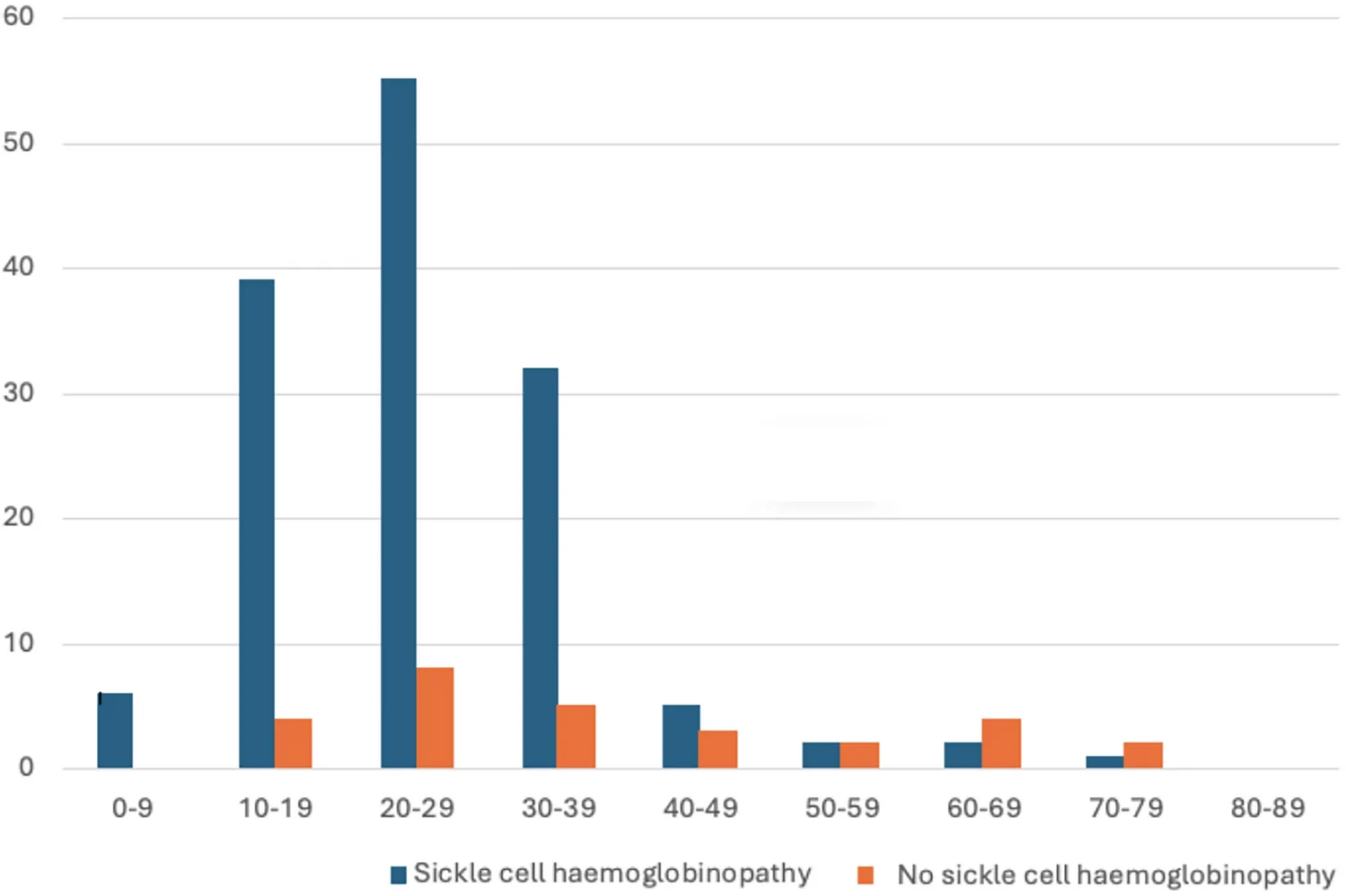
Bar chart of age at diagnosis in patients with sickle cell haemoglobinopathy (either sickle cell trait or disease) versus no sickle cell haemoglobinopathy

Age at presentation was documented in 188 cases, ranging from 6 to 72 years. The mean age was 27.5 (± 14.1) years. When comparing those with SCH (*n* = 168) versus those without (*n* = 20), the age at presentation was younger: 27.3 (± 13.7) years versus 34.1 (± 19.1) years, respectively (*p* = 0.001) (Fig. 2).

Information on gender was available in 221 cases. 160 were male (72.4%); and 61 were female (27.6%). There was a male preponderance among both patients with and without sickle cell haemoglobinopathy.

### Clinical features

Clinical features are described in Table 1. The clinical presentation was documented in 183 cases (81.7%). The most common presentations were haematuria or abdominal/flank pain, present in 59.6% (*n* = 109) and 54.1% (*n* = 99) of patients, respectively. Only a single patient was reported as presenting with the classic triad of RCC symptoms. However, 88% (*n* = 161) experienced at least one of haematuria, abdominal/flank pain, or palpable abdominal mass. Systemic symptoms of malignancy (e.g. fever or weight loss) were present in 24.0% (*n* = 44), with those patients having an increased likelihood of Stage IV disease at presentation (relative risk 1.36; 95% CI 1.10–1.69). RMC was discovered incidentally in 5 patients (2.7%).

Patients were grouped according to the AJCC prognostic stage groups, based upon the TNM staging system for RCC [5]. 17 patients (8.4%) were Stage I–II at presentation; 42 (20.7%) were Stage III, with a T3 tumour and/or retroperitoneal lymphadenopathy; and 144 (70.9%) were stage IV, indicating the presence of either distant metastases or a locally invasive T4 tumour. Patients with SCH were more likely to present at an advanced stage (*p* = 0.007).

Information on the presence of metastatic disease at the time of presentation was available for 159 (71%) patients at the time of diagnosis (Table 2). 24 patients had no nodal or distant disease. Of the 135 *with* retroperitoneal nodal or distant metastases, retroperitoneal lymph nodes were the most involved site (*n* = 115 (85.2%)). Lung, liver, supra-diaphragmatic lymph nodes, and bone were also commonly involved (*n* = 60 (44.4%), *n* = 38 (28.1%), *n* = 30 (22.2%) and n = 27 (20.0%), respectively). For 65 patients, no detailed documentation on sites of disease at presentation was available, although 42 of those were known to be Stage IV.

**Table 2:** Distribution of nodal and distant metastases seen in patients with medullary renal cancer at initial presentation, and over the follow-up period

	At presentation		Over follow-up course	
Total with nodal or distant metastases	135		177	
Retroperitoneal lymph nodes	115	85.2%	151	85.3%
Non-regional lymph nodes	30	22.2%	36	20.3%
Lung	60	44.4%	107	60.5%
Liver	38	28.1%	61	34.5%
Adrenal	13	9.6%	21	11.9%
Pleura	14	10.4%	18	10.2%
Brain	4	3.0%	11	6.2%
Bone	27	20.0%	55	31.1%
Other	20	14.8%	30	16.9%

Information on sites of metastatic disease over the course of follow-up was available in 194 patients (Table 2). Over the course of the follow-up, 177 developed nodal or distant metastatic disease. 17 patients remained free from nodal involvement and metastases throughout their follow-up period. For 28 patients, no specific sites of metastatic involvement were documented (although *n* = 14 of those were Stage IV at presentation). 151 (85.3%) patients had retroperitoneal lymph node involvement. Lung (*n* = 107 (60.5%)), liver (*n* = 61 (34.5%)) and bone (*n* = 55 (31.1%)) were common sites of metastatic involvement. Non-regional lymph nodes and adrenal metastases were all present in > 10%. 13 patients developed local recurrence in the nephrectomy bed out of the 130 that underwent nephrectomy (10%).

Unusual metastatic sites included: female pelvic organs [6–8]; skin [9–11]; pulmonary lymphangitis [4, 12]; orbit [13–15]; mesentery or peritoneum [16–18]; breast [19, 20]; subcutaneous or muscular deposits [21, 22]; parotid gland, thyroid, cardiac, pancreas, spleen, cross-renal, and bladder [7]. In many cases, there was rapid development of metastases in patients initially staged I–III.

Information on management was documented in 183 cases. In total, 130 (71%) patients underwent nephrectomy (two of which were partial nephrectomies), 109 (59.6%) received chemotherapy; and 33 (18%) received immunotherapy or targeted therapy after 2011. 17 (9.3%) patients underwent metastasectomy and 16 (8.7%) received radiotherapy. 12 (6.6%) received only supportive palliative care. Of the 108 patients that had stage IV disease at diagnosis and documented management, 75 (71%) received chemotherapy, 68 (63%) underwent nephrectomy, 20 (18.5%) received immunotherapy, 12 (11.1%) received radiotherapy, 9 (8.3%) underwent a metastasectomy, and 7 (6.5%) received supportive care only.

**Fig. 3:**
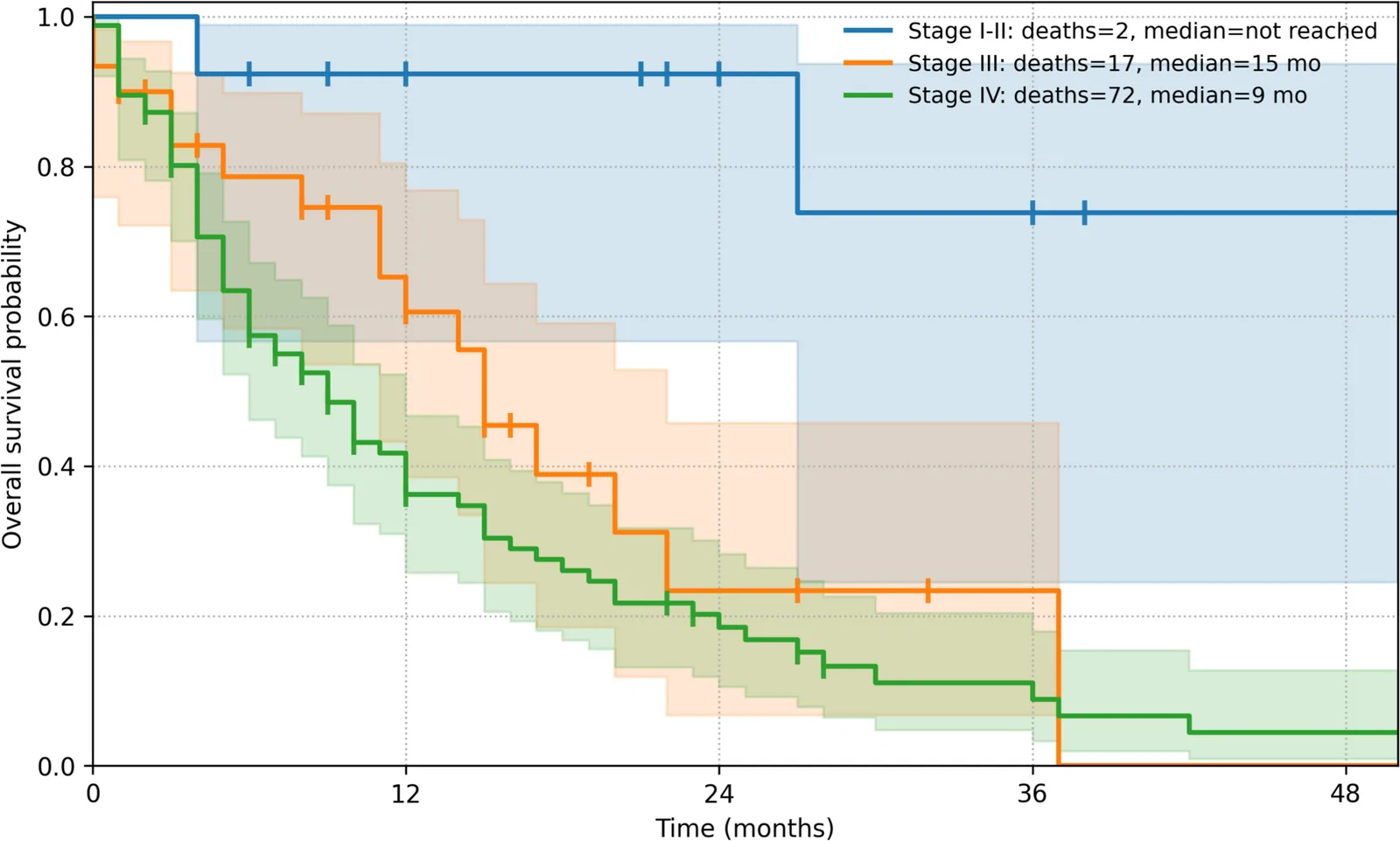
Kaplan–Meier survival plot by tumour stage at presentation

Survival information was available for 153 patients, with death reported in 112, and survival at the end of follow-up in 41. When comparing OS, grouped by Stage at presentation (*I–II* versus *III* versus *IV*—Table 3), there was a significant difference between the groups (p = < 0.001), with OS in Stage IV patients being 36%, 18% and 9% at 1, 2 and 3 years, respectively (Fig. 3**, **Table 3).

**Table 3:** Survival rates stratified by stage at diagnosis over 12, 24, and 36 months from diagnosis

Stage at diagnosis	12-mo OS, % (95% CI)	No. at risk at 12-mo*	24-mo OS, % (95% CI)	No. at risk at 24-mo*	36-mo OS, % (95% CI)	No. at risk at 36-mo*
Stage I-II (*n* = 13)	92.3 (56.6–98.9)	10	92.3 (56.6–98.9)	6	73.8 (24.5–93.7)	4
Stage III (*n* = 30)	60.5 (38.5–76.8)	14	23.4 (6.7–45.8)	3	23.4 (6.7–45.8)	1
Stage IV (*n* = 86)	36.2 (25.7–46.7)	30	18.4 (10.5–28.3)	12	8.8 (3.2–17.9)	5

*The number at risk is those alive and non-censored at the end of each 12-month period

### Radiological findings

Information on the imaging studies used in initial workup was available for 143 patients. 98.6% underwent CT (*n* = 141); US 24.5% (*n* = 35); 10.5% had MRI (*n* = 15); ^18^F-FDG PET/CT 6.3% (*n* = 9); and excretory urography 7.7% (*n* = 11). Small numbers of patients also underwent retrograde studies, digital subtraction angiograms, bone scans, and a glucoheptonate scan. Of the 35 undergoing US, imaging findings were described in 29 cases; the mass was not appreciated in 9 (31%).

For 189 patients the side of the primary tumour was documented: tumours were more likely to be right-sided (*n* = 122, 64.6%, *p* = < 0.001). One patient had RMC in the right side of a horseshoe kidney [23]. When grouped by sickle cell status, 71.1% (*n* = 96/135) of SCH-related cases were right-sided, compared to 59.3% of non-SCH cases (*n* = 16/27), which was not a statistically significant difference (*p* = 0.32). The size of the primary tumour was documented in 127 cases, ranging from 1.8 cm to 19.0 cm. The mean tumour size was 6.4 ± 2.9 cm. There was no difference in the size of the tumour between patients with and without SCH (6.5 cm versus 6.3 cm, respectively, *p* = 0.46). The tumour was centred on the upper pole in 40 (41.7%), the lower pole in 33 (34.3%), and the interpolar region in 23 (24%). 107 tumours were described as endophytic; none were predominantly exophytic.

The amount of radiological description and imaging used in figures varied considerably between articles. The primary tumours were poorly defined or infiltrative in 65 cases, and well-defined in 16. The tumour was seen as hypo-enhancing in 71 and avidly enhancing in 4. Necrosis was described in 29 tumours. Caliectasis was present in 26, with frank hydronephrosis in 16 of those. Calcification (*n* = 1) and haemorrhage (*n* = 8) were rare. Renal vein invasion was described in 17 and encasement of the vascular pedicle or IVC/aorta by either tumour or retroperitoneal nodal mass was seen in 12.

Two tumours were described as cystic, including one classified as Bosniak III [24] and one 4.5 cm “benign-appearing” cyst on first presentation, which enlarged to 5.6 cm over 6 months with malignant cells found at decortication [25].

## Discussion

Our review identified 224 distinct cases of RMC. The disease usually presents at a late stage, and in patients with nodal or distant metastases, the prognosis is extremely poor. Most patients are of Black ethnicity, or have SCH, and in the latter the tumour presents at a younger age and is most commonly right-sided.

Radiologically, the primary tumour is almost universally endophytic, centred on the renal medulla. It typically enhances to a lesser degree than the renal cortex and has an infiltrative border. Metastases are commonly present at diagnosis, particularly retroperitoneal lymphadenopathy, but a wide range of metastatic sites have been reported.

### Limitations

There are inherent challenges in performing a review of RMC. Data either come from case reports, small series, or summaries from larger national cancer databases. Care has been taken over the course of this review to avoid duplication, so that each patient is only included once. As we have desired detailed information to build a holistic picture of this rare condition, where there has been a risk of duplication, single cases or small series with more detail have been preferred leading us to exclude some larger studies with more cases. Some previously published review articles have not made allowance for this duplication and have included individual cases that are highly likely to also be included in other summative work published from the same institutions or in the US national tumour registry studies [26].

Published articles come from the fields of Urology, Oncology, Haematology, Pathology, and Radiology with differing content included in the write-up. As more cases have been published, recent studies are likely to suffer from publication bias, being submitted and accepted for publication based on novel metastatic sites or a promising response to therapy. RMC is also likely underdiagnosed as there is considerable overlap of histopathological features with collecting duct carcinoma and other aggressive renal tumours, urothelial carcinoma and rare metastatic germ cell tumours [3].

In assessment of radiological features, the description of the tumour in the articles has been taken as correct, and the published figures have been assumed to be representative. The absence of a radiological feature being reported does not conclusively mean it is not present, and as such it is only possible to say which features are commonly described, and not to give the prevalence of those findings. In many cases, the articles are published either before death or before an adequate follow-up period, meaning the sites of metastases described may be an underestimate. Additionally, whilst we recorded the treatments given to show heterogeneity, we did not capture the sequence or therapeutic intent. As the treatments given varied extensively and the outcomes are from predominantly case reports/small series, meaningful survival analyses or robust assessments of treatment benefit are precluded.

Despite these difficulties, we have found merit in summarising the findings of this rare disease, and of using case reports, which contain more detail on each patient story than can be covered in national database summaries.

### Epidemiology and aetiology

Although SCT has frequently been present in those with RMC, it is not present in *all* cases and these can be regarded as subtypes of SMARCB1-deficient RCC with medullary-like features. However, loss of expression of SMARCB1/INI1 *is* ubiquitous regardless of sickle cell status [2, 27]. Normally SMARCB1/INI1 encodes a component of the switch/sucrose nonfermentable complex, which is required for several cell functions, and acts as a tumour suppressor [28]. However, it is important to note that secondary SMARCB1-deficiency has been described in clear cell RCC with sarcomatoid differentiation, collecting duct carcinoma with secondary SMARCB1 loss or fumarate hydratase (FH)-deficient RCC—they should not be classified as RMC [29].

A model has been proposed by Msaouel et al. linking SCH with SMARCB1/INI1 loss [30]: in brief, to facilitate the concentration of urine, the renal medulla is an extremely hypoxic and hypertonic environment, which can lead to sickling of erythrocytes, even in patients with SCT. Sickling in the renal medulla leads to microinfarctions, compounding the already hypoxic microenvironment, leading to DNA damage. This also accounts for the presence of sickled erythrocytes in nephrectomy specimens, including in patients with SCT.

Also outlined by Msaouel and colleagues, it is *all* sickle cell haemoglobinopathies that are associated, not just SCT [30]. This is evidenced by the similar prevalence of RMC in SCT and SCD; it is simply that SCD is far less common (approximately 8.3% of African Americans have SCT but only 0.15% have SCD) [30]. The estimated prevalence of RMC is approximately 1/39,000 for patients with SCT in the USA [31].

It has therefore been proposed that SCH, and the hypoxia it causes, acts as one of the insults in the ‘two-hit hypothesis’ of cancer development [32]; this would be supported by our finding that RMC occurs at a younger age in patients with SCT (27.3 versus 34.1 years, *p* = 0.001).

Our data show that patients with SCH presented at a more advanced stage than those without. This may reflect delayed diagnosis as RCC is unexpected in younger patients, and presenting symptoms may be attributed to alternative or non-malignant sickle cell-related causes.

RMC more commonly affected the right kidney in our sample (122 of 189 cases (64.6%)); however, when stratified by SCH status, in the non-SCH cohort, cases were more evenly distributed between right and left (59.3% v 40.7%), whereas in those with SCH, 71.1% were right-sided. This initially appears counterintuitive as the left renal vein is longer and can be compressed between the aorta and superior mesenteric artery, leading to venous stasis. A theory for the increased right-sidedness is that flow is inversely proportional to the length of a vessel, and that as the right renal artery is longer, with consequently lower pressures, it predisposes to hypoxia on that side [30]. This has also been postulated as the reason for cocaine-induced renal infarction being far more common in the right kidney [33].

### Radiological findings

**Fig. 4:**
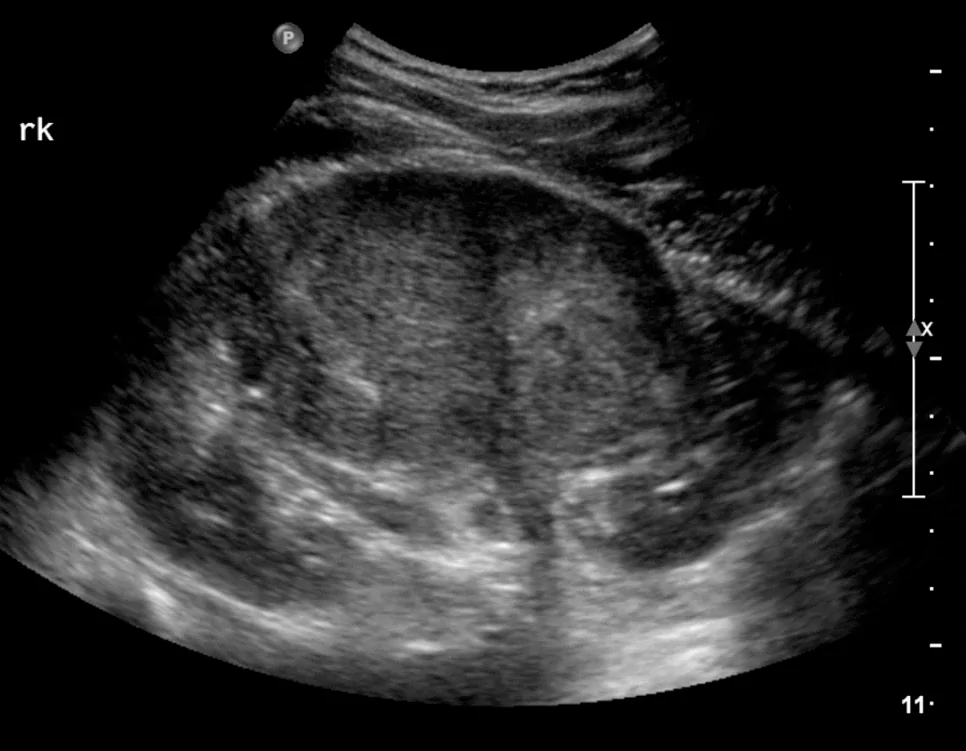
20-year-old Black male with sickle cell trait presenting with haematuria. Ultrasound scan showing an endophytic 8 cm mass in the interpolar region of the right kidney, compressing the collecting system

**Fig. 5:**
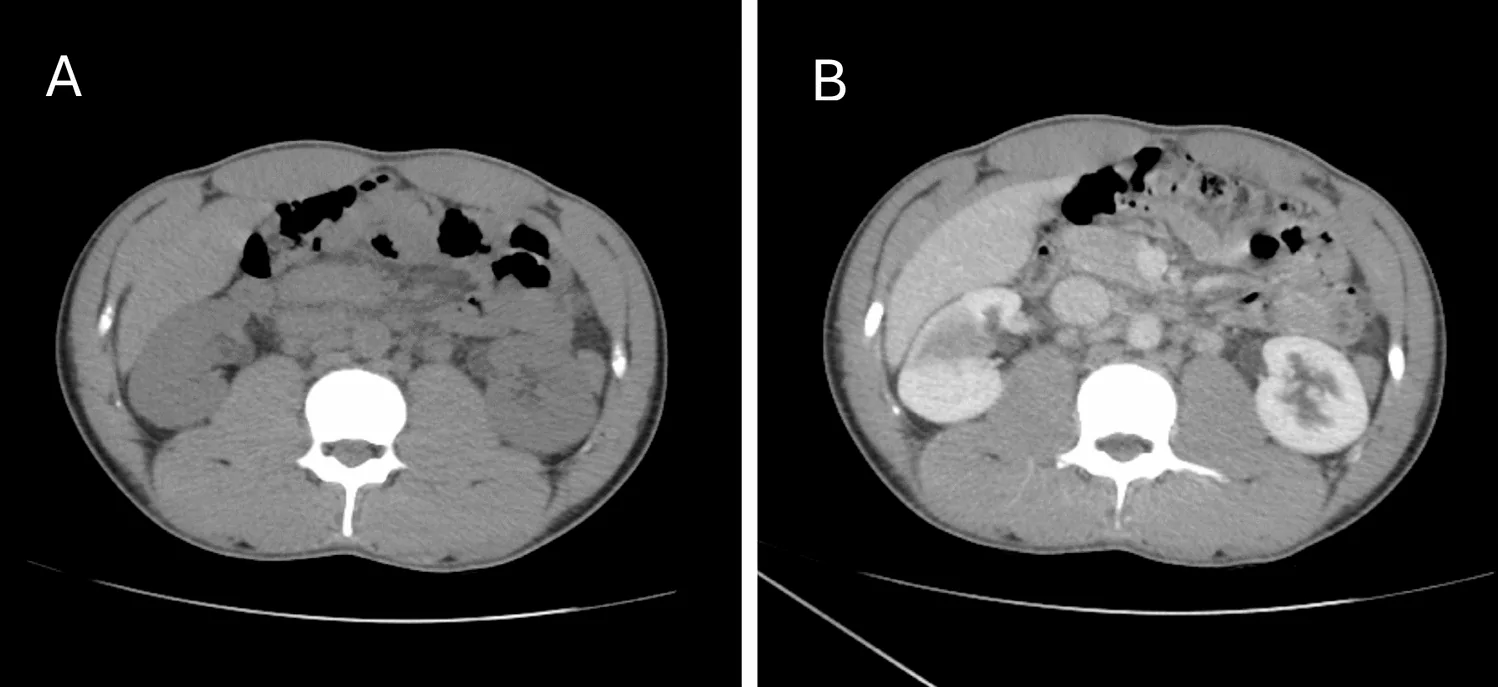
23-year-old Black male with sickle cell trait presenting with haematuria. After a normal ultrasound, he re-presented one month later with haemoptysis and chest pain and was diagnosed with a pulmonary embolus. Enlarged lymph nodes were noted above and below the diaphragm. An 18F-FDG PET/CT did not detect the primary tumour. A) Non-contrast-enhanced and B) contrast-enhanced CT scans show a 3.2 cm hypoenhancing right interpolar renal mass protruding into the collecting system, with retroperitoneal lymphadenopathy

Almost all patients underwent CT during their diagnostic workup. As would be expected given the young age at presentation, US was performed in many (Fig. 4). However, in 31% of cases where the findings were documented, the tumour was not identified, presumably due to the endophytic, poorly defined nature of the RMC. As tumours are large at presentation (on average 6.4 cm) and central in location, arising from the medulla, the kidney is often seen as enlarged on unenhanced CT (CT KUB) or US. Given the normal reniform shape is usually preserved, there can be challenges identifying the tumour on an unenhanced CT (Fig. 5), which suggests that performing only US or low-dose CT of the urinary tract in the workup of haematuria, even in younger patients, is inadequate.

**Fig. 6:**
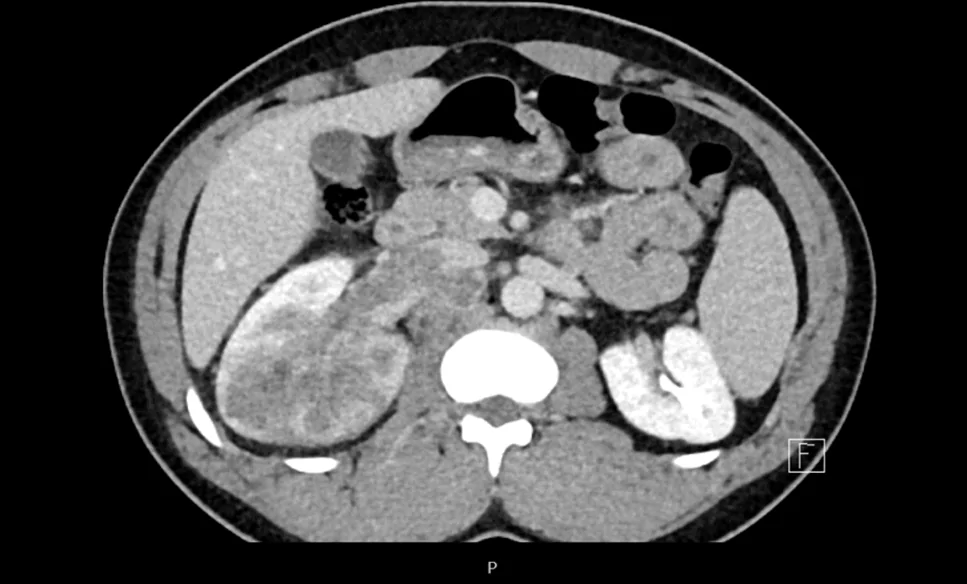
29-year-old Black female with sickle cell trait presenting with haematuria. Axial contrast-enhanced venous-phase CT scan shows an infiltrative tumour replacing the right kidney, extending into the renal vein and inferior vena cava, with retroperitoneal nodal disease. There were also metastases to the lungs, liver, bone, and supradiaphragmatic lymph nodes (not shown)

**Fig. 7:**
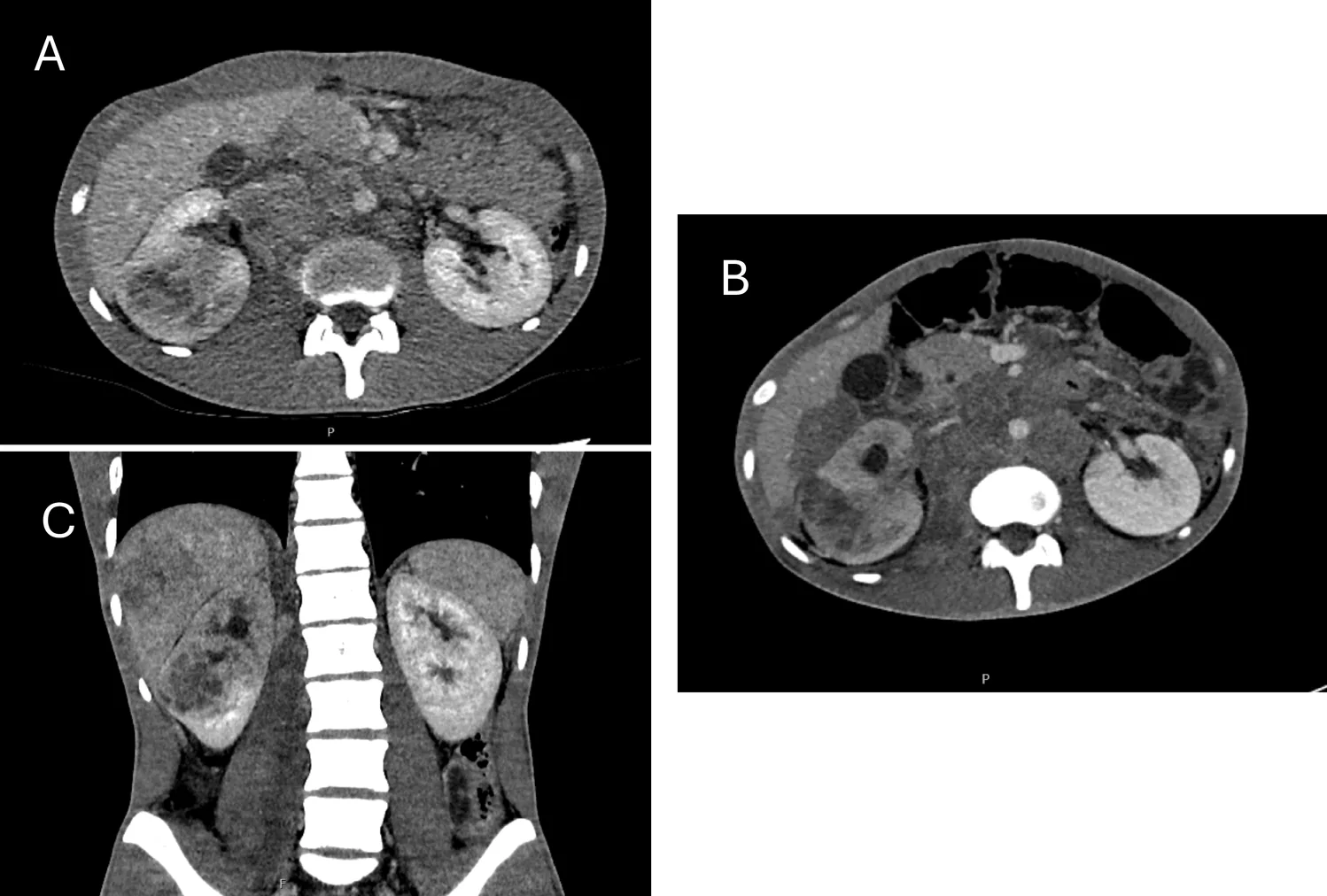
22-year-old Black male without sickle cell trait presenting with abdominal pain. **A**, **B** Axial and **C** coronal contrast-enhanced venous-phase CT scans show a largely necrotic right renal tumour with upper pole caliectasis, retroperitoneal lymph nodes encasing the aorta and inferior vena cava, and a liver metastasis. Lung metastases were also present (not shown)

**Fig. 8:**
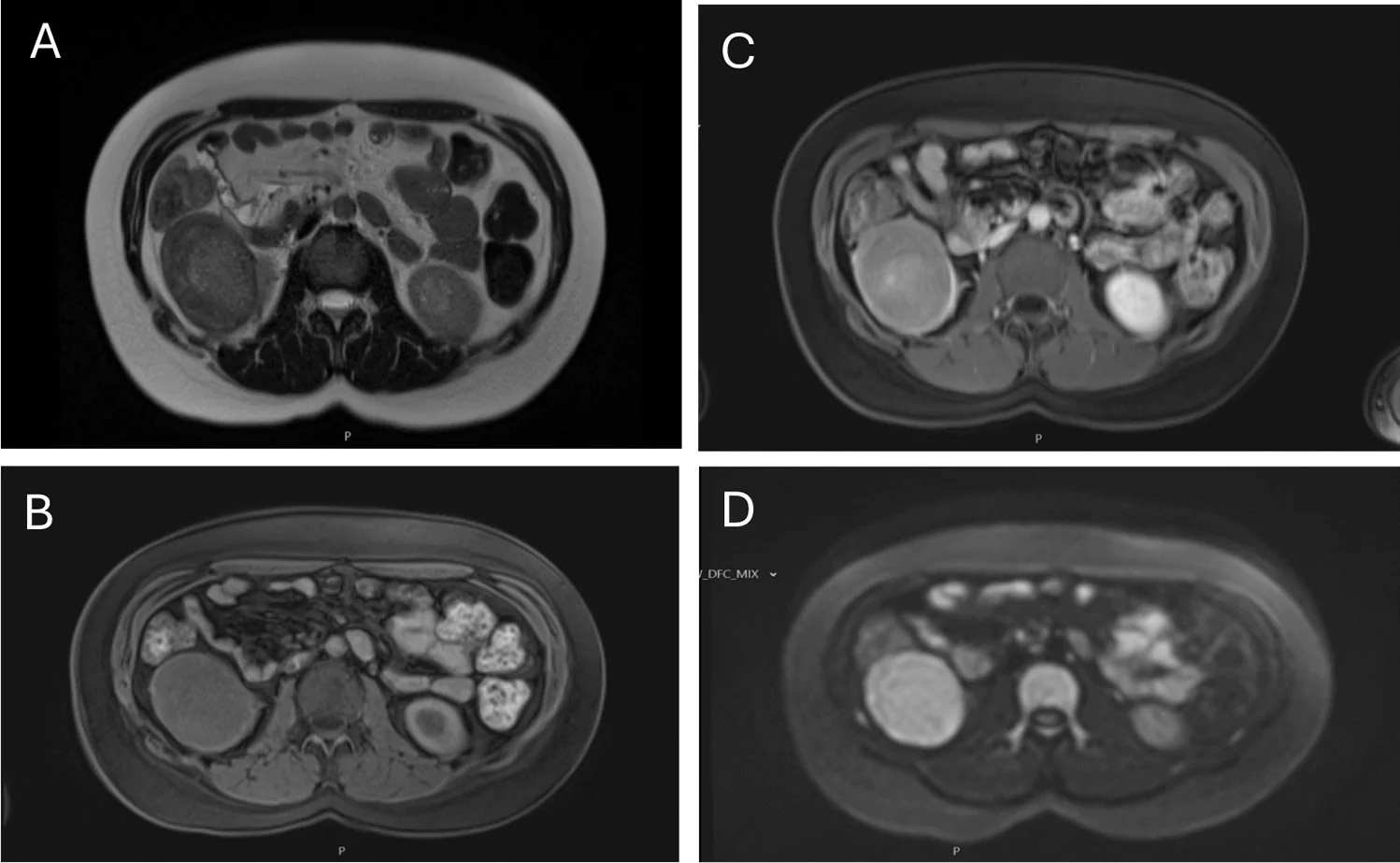
29-year-old Middle Eastern female without sickle cell trait presenting with abdominal pain. **A** Axial T2-weighted (T2w), **B** fat-saturated non-contrast-enhanced T1-weighted (T1w), **C** contrast-enhanced T1-weighted, and **D** b800 diffusion-weighted MRI images show a well-defined exophytic right renal mass. It is heterogeneous on T2w, isointense on T1w, and hypoenhancing relative to the cortex. The mass effaces the renal sinus fat and lower pole calyces, with restricted diffusion (ADC map not shown)

On contrast-enhanced CT, when coupled with the central location, RMC can be suggested given its differential enhancement characteristics compared with other subtypes of RCC. RMC enhances to a lesser degree than the cortex in 94.7% (*n* = 71/75) (Figs. 5, 6, 7, 8). Clear cell is the most common subtype of RCC, but is typically hyper-vascular and expansile, centred on the renal cortex; peripheral enhancement and central necrosis are common [34]. Papillary RCCs represent approximately 10% of RCC, and although hypo-enhancing, these are centred on the cortex, enhance uniformly (especially when small), and are well-defined. Chromophobe RCCs also tend to be centred on the cortex and are well-defined but can have a wide range of appearances; classically homogeneously hypo-enhancing when small and with a central scar or necrosis when larger. Although calcification is not common, seen in approximately 14% [35], this is still much higher than in RMC, described in only 1 case in our review.

The tumour most like RMC is collecting duct carcinoma (CDC), which is equally rare and centred on the renal medulla. It has an infiltrative growth pattern radiologically and histologically and enhances to a lesser degree than the normal cortex. Whilst these are all also features of RMC, CDC commonly has an exophytic component (seen in 39–59% of cases) [36, 37] and a cystic component is present in 35–50% [36, 37]. The tumour size at presentation is similar: 6.4 cm for RMC versus 6.9 and 7.7 cm in two series of CDC [36, 37]. Although there is considerable overlap in imaging features, the typical age at presentation differs (mean 27.5 years for RMC versus 53–56 years in CDC [36, 37]) and there is no link to haemoglobinopathies. It has been proposed that many cases of CDC, particularly those prior to 1995, could have been mis-diagnosed RMC [26]. There is considerable histological overlap, but loss of expression of SMARCB1/INI1 is a potential differentiator, seen in all cases of RMC but in less than 5% of CDC with secondary SMARCB1 loss [28]. Renal vein invasion was described in 17 cases (Fig. 6) and encasement of the renal vascular pedicle or IVC by nodal mass is also not uncommon (Fig. 7).

MRI is mostly used as a problem-solving tool in assessment of renal masses and was reported to have been performed in 11% of cases in our series. We found RMC to be of intermediate/low T2w signal relative to the normal adjacent cortex, intermediate T1w signal, and to enhance heterogeneously to a lesser degree than the adjacent cortex. The tumour displayed restricted diffusion (Fig. 8). In three cases, Blitman et al. found the tumour margins equally well demonstrated on CT and MRI [38]. Internal haemorrhage and necrosis are also better seen on MRI, and calcification less well, although there is no clinical relevance to this [39].

### Metastatic profile, management and survival data

Retroperitoneal lymph node involvement has been reported at presentation in 13–32% of RCC [40], however in our cohort of RMC 72.3% had retroperitoneal lymph nodal disease. This is likely to be an underestimate as many other cases were either Stage III or Stage IV at presentation, without documentation of exact metastatic sites. Including all RCC subtypes, 25% were historically Stage IV at diagnosis [41], although this is likely to be decreasing as increasingly renal tumours are diagnosed early and incidentally [42]. Our study found Stage IV disease in 70.9% at presentation in RMC. Overall, a wide range of metastatic sites have been reported, including those responsible for the clinical presentation.

Beckermann et al. have tried to provide some standardisation in the management of patients with RMC. They suggest nephrectomy with retroperitoneal lymph node dissection (RPLND) and consideration of adjuvant therapy for Stage I–III disease, with close surveillance every 2–4 months [2]. For metastatic disease, they recommend cytoreductive nephrectomy with RPLND (if performance status and disease distribution permit) followed by platinum-based combination chemotherapy or clinical trial enrolment, again with close follow-up. In our study, 17 cases that had Stage I–II disease all underwent nephrectomy and 23.5% (*n* = 4) received chemotherapy. It should be noted that there are no reported trials of adjuvant therapy in RMC and limited evidence for efficacy of checkpoint inhibitors in RMC and thus no high-level evidence for offering this routinely. Of 108 cases that had Stage IV disease, 71% (*n* = 75) received chemotherapy and 63% (*n* = 68) underwent a nephrectomy.

A full review of management is outside the scope of this article and data are currently limited. However, there are increasing numbers of reports of individual cases deriving benefit from other approaches such as targeted therapy and immunotherapy. In our review, of those who had documented management, 18% received immunotherapy (11.7% of those with Stage I–II, 17.6% stage III, and 18.5% stage IV).

Overall survival was poor in RMC. Survival information was available in 153 cases, with death reported in 112. Many patients were alive but with progressive metastatic disease at the time of case report submission. Survival analyses should be interpreted cautiously from this data, with small numbers at risk and wide confidence intervals by 2- and 3-years (OS). OS in RMC patients with Stage IV disease was 36% at 1 year and 9% at 3 years, which is less than the 1- and 5-year OS rates seen in RCC overall (45% and 20%, respectively, from 2021 SEER data [43]), albeit with overlapping and close confidence intervals. Contrastingly, the OS rates for Stage III disease appear much poorer in RMC (1- and 3-year OS was 61% and 23% for RMC versus 1-year OS of 92% and 5-year OS of 74% in RCC overall) [43]. The high rates of Stage IV disease at presentation and poor survival rates, even in those without distant metastases at diagnosis, confirm the aggressive nature of the disease seen anecdotally in clinical practice (Fig. 7).

## Conclusion

The rarity of the disease, unusual age of presentation, and overlapping signs and symptoms with other aspects of sickle cell haemoglobinopathies make early diagnosis of RMC a challenge. The endophytic, infiltrative radiological appearances can make identifying the tumour difficult on US and non-contrast CT, suggesting these investigations alone are insufficient in assessment of haematuria, even in younger patients, and that contrast-enhanced CT or MRI should be considered. Patients diagnosed with RMC require aggressive management and close follow-up, as the disease can progress rapidly, even in those with smaller tumours, clear surgical margins, and an absence of metastatic disease at the time of resection.

## Supplementary Information

Below is the link to the electronic supplementary material.

Supplementary file1 (DOCX 27 KB)

Supplementary file2 (DOCX 27 KB)

## Data Availability

No datasets were generated or analysed during the current study.

## Glossary

CDC: Collecting duct carcinoma
RMC: Renal medullary carcinoma
SCD: Sickle Cell Disease
SCH: Sickle cell haemoglobinopathy
SCT: Sickle cell trait
