# Supplementary material for: Renal medullary carcinoma: a systematic review of clinical and radiological findings
Source: Int Urol Nephrol. 2026 Apr 9;58(9):3407–18. doi: 10.1007/s11255-026-05113-4 (PMC13506601; doi:10.1007/s11255-026-05113-4)
Supplement: Supplementary file 1 [file 11255_2026_5113_MOESM1_ESM.docx]

**SUPPLEMENTARY MATERIAL**

1. Popov SD, Sebire NJ, Pritchard-Jones K, Vujanic GM. Renal tumors in children aged 10–16 years: a report from the United Kingdom Children's Cancer and Leukaemia Group. Pediatr Blood Cancer. 2014;61(4):593-598.
2. Raman SP, Johnson PT, Allaf ME, Netto G, Fishman EK. Chromophobe renal cell carcinoma: multiphase MDCT enhancement patterns and morphologic features. AJR Am J Roentgenol. 2013;201(6):1268-1276.
3. Johnson RP, Krauland K, Owens NM, Peckham S. Renal medullary carcinoma metastatic to the scalp. Am J Dermatopathol. 2011;33(1):e11-e13.
4. Shetty A, Matrana MR. Renal medullary carcinoma: a case report and brief review of the literature. Ochsner J. 2014;14(2):270-275.
5. Amjad AI, Al-Saanna GA, Matin SF. Renal medullary carcinoma: case report of an aggressive malignancy with near-complete response to dose-dense methotrexate, vinblastine, doxorubicin, and cisplatin chemotherapy. Case Rep Oncol Med. 2014;2014:615895.
6. Brown JM, Berkey BD, Brooks JA. Discovery of a renal medullary carcinoma in an adolescent male with sickle cell trait by Tc-99m methylene diphosphonate bone scintigraphy. Clin Nucl Med. 2008;33(12):896-900.
7. Ronnen EA, Kondagunta GV, Motzer RJ. Medullary renal cell carcinoma and response to therapy with bortezomib. J Clin Oncol. 2006;24(9):e14.
8. Qi J, Shen PU, Rezuke WN, et al. Fine needle aspiration cytology diagnosis of renal medullary carcinoma: a case report. Acta Cytol. 2001;45(5):735-739. doi:10.1159/000328296.
9. Carlo MI, Chaim J, Patil S, et al. Genomic characterization of renal medullary carcinoma and treatment outcomes. Clin Genitourin Cancer. 2017;15(6):e987-e994.
10. Shi Y, Raju S, Siegal GP. Fine needle aspiration cytology diagnosis of renal medullary carcinoma: a case report. Acta Cytol. 2001;45(5):773-777.
11. Alvarez O, Rodriguez MM, Jordan L, Sarnaik S. Renal medullary carcinoma and sickle cell trait: a systematic review. Pediatr Blood Cancer. 2015;62(10):1694-1699.
12. Rathmell WK, Monk JP. High-dose-intensity MVAC for advanced renal medullary carcinoma: report of three cases and literature review. Urology. 2008;72(3):659.e3-659.e5.
13. Ellis CL, Burroughs F, Michael CW, Li QK. Cytology of metastatic renal medullary carcinoma in pleural effusion: a study of two cases. Diagn Cytopathol. 2009;37(11):803-808.
14. Watanabe IC, Billis A, Guimarães MS, et al. Renal medullary carcinoma: report of seven cases from Brazil. Mod Pathol. 2007;20(9):914-920. doi:10.1038/modpathol.3800934.
15. Rao P, Tannir NM, Tamboli P. Expression of OCT3/4 in renal medullary carcinoma represents a potential diagnostic pitfall. Am J Surg Pathol. 2012;36(4):583-588. doi:10.1097/PAS.0b013e3182417d78.
16. Khabir A, Mnif L, Krichen Makni S, et al. Renal medullary carcinoma: a case report. Cancer Radiother. 2006;10(2):134-136.
17. Schaeffer EM, Guzzo TJ, Furge KA, et al. Renal medullary carcinoma: molecular, pathological, and clinical evidence for treatment with topoisomerase-inhibiting therapy. BJU Int. 2010;106(1):62-65.
18. Beckermann KE, Sharma D, Chaturvedi S, et al. Renal medullary carcinoma: establishing standards in practice. J Oncol Pract. 2017;13(7):414-421.
19. Miller DL, Ribeiro EA, Roy-Chowdhuri S, et al. Renal medullary carcinoma involving serous cavity fluids: a cytomorphologic study of 12 cases. J Am Soc Cytopathol. 2021;10(2):187-196. doi:10.1016/j.jasc.2020.05.016.
20. Hakimi AA, Koi PT, Milhoua PM, et al. Renal medullary carcinoma: the Bronx experience. Urology. 2007;70(5):878-882. doi:10.1016/j.urology.2007.06.1124.
21. Davidson AJ, Choyke PL, Hartman DS, Davis CJ. Renal medullary carcinoma associated with sickle cell trait: radiologic findings. Radiology. 1995;195(1):249-252. doi:10.1148/radiology.195.1.7892499.
22. Maroja Silvino MC, Moniz CMV, Piotto GHM, et al. Renal medullary carcinoma response to chemotherapy. Reports in Toxicology. 2013;1:e44. doi:10.4081/rt.2013.e44.
23. Coogan AS, Davis CJ. Renal medullary carcinoma in patients with sickle cell trait. Urology. 1998;51(5):799-803. doi:10.1016/S0090-4295(98)00104-6.
24. Wartchow EP, Trost BA, Tucker JA, Albano EA, Mierau GW. Renal medullary carcinoma: ultrastructural studies may benefit diagnosis. Ultrastruct Pathol. 2008;32(6):252-256.
25. Assad L, Resetkova E, Oliveira VL, et al. Cytologic features of renal medullary carcinoma. Cancer. 2005;105(1):28-34.
26. Carden MA, Perdahl-Wallace E, Greenberg J, McCarty JM. Autologous stem-cell transplant for metastatic renal medullary carcinoma. Pediatr Blood Cancer. 2018;65(9):e27267. doi:10.1002/pbc.27267.
27. Sodji QA, Davis CJ. Predictive role of PD-L1 expression in the response of renal medullary carcinoma to nivolumab. Front Oncol. 2017;7:171. doi:10.3389/fonc.2017.00171.
28. Carter SA, Walker AN. Renal medullary carcinoma and sickle cell trait: a push for early diagnosis and intervention report of two cases. J Natl Med Assoc. 2017;109(1):63-65. doi:10.1016/j.jnma.2016.08.006.
29. Gangireddy VGR, Liles GB, Sostre GD, Coleman T. Response of metastatic renal medullary carcinoma to carboplatinum and paclitaxel chemotherapy. Clin Genitourin Cancer. 2012;10(2):134-139. doi:10.1016/j.clgc.2012.01.003.
30. Strouse JJ, Spevak M, Mack AK, et al. Significant responses to platinum-based chemotherapy in renal medullary carcinoma. Pediatr Blood Cancer. 2005;44(4):407-411.
31. Yang X, Wang L, Zhang Y. Gene expression profiling of renal medullary carcinoma. Cancer. 2004;101(10):2345-2353. doi:10.1002/cncr.20049.
32. Figenshau RS, Basler JW, Ritter JH, et al. Renal medullary carcinoma. J Urol. 1998;159(3):711-713.
33. Patel K, Livni N, Macdonald D. Renal medullary carcinoma, a rare cause of haematuria in sickle cell trait. Br J Haematol. 2006;132(1):1. doi:10.1111/j.1365-2141.2005.05795.
34. Pickhardt PJ, Siegel CL, McLarney JK. Collecting duct carcinoma of the kidney. AJR Am J Roentgenol. 2001;176(3):627-633.
35. Levi Sandri GB, Spoletini G, Lai Q, et al. Cystic presentation of a renal medullary carcinoma in a young woman. Urologia. 2015;82(2):124-126.
36. Mariño-Enríquez A, Ou WB, Weldon CB, et al. ALK rearrangement in sickle cell trait-associated renal medullary carcinoma. Genes Chromosomes Cancer. 2011;50(3):146-153. doi:10.1002/gcc.20839.
37. Leitão VA, da Silva W Jr, Ferreira U, et al. Renal medullary carcinoma. Case report and review of the literature. Urol Int. 2006;77(2):184-186. doi:10.1159/000093918.
38. Khan A, Thomas N, Costello B, et al. Renal medullary carcinoma: sonographic, computed tomography, magnetic resonance, and angiographic findings. Eur J Radiol. 2000;35(1):1-7. doi:10.1016/s0720-048x(99)00101-1.
39. Stahlschmidt J, Cullinane C, Roberts P, Picton SV. Renal medullary carcinoma: prolonged remission with chemotherapy, immunohistochemical characterisation and evidence of bcr/abl rearrangement. Med Pediatr Oncol. 1999;33(6):551-557. doi:10.1002/(sici)1096-911x(199912)33:6<551::aid-mpo5>3.0.co;2-5.
40. Baig MA, Lin YS, Rasheed J, Mittman N. Renal medullary carcinoma. J Natl Med Assoc. 2006;98(7):1171-1174.
41. Colombo P, Smith SC, Massa S, et al. Unclassified renal cell carcinoma with medullary phenotype versus renal medullary carcinoma: lessons from diagnosis in an Italian man found to harbor sickle cell trait. Urol Case Rep. 2015;3(6):215-218. doi:10.1016/j.eucr.2015.07.011.
42. Warren KE, Gidvani-Diaz V, Duval-Arnould B. Renal medullary carcinoma in an adolescent with sickle cell trait. Pediatrics. 1999;103(2):E22. doi:10.1542/peds.103.2.e22.
43. Wesche WA, Wilimas J, Khare V, Parham DM. Renal medullary carcinoma: a potential sickle cell nephropathy of children and adolescents. Pediatr Pathol Lab Med. 1998;18(1):97-113.
44. Thomas C, Khan A, Khrishnan H, Das G. Renal medullary carcinoma is a diagnosis worth considering: case report. World J Oncol. 2010;1(4):176-178. doi:10.4021/wjon222w.
45. Hoenig JD, Husain AN, Waters BW. Pathologic quiz case: gross hematuria in a 20-year-old black woman with sickle cell trait. Arch Pathol Lab Med. 2002;126(5):627-629. doi:10.5858/2002-126-0627-PQCGHI.
46. Chatelain D, de Pinieux G, Slama J, et al. Renal medullary carcinoma, a new clinico-pathological entity. Ann Pathol. 1999;19(4):320-324.
47. Larson DM, Gilstad CW, Manson GW, Henry MR. Renal medullary carcinoma: report of a case with positive urinary cytology. Diagn Cytopathol. 1998;18(4):276-279. doi:10.1002/(sici)1097-0339(199804)18:4<276::aid-dc5>3.0.co;2-a.
48. Herring JC, Schmetz MA, Digan AB, Young ST, Kalloo NB. Renal medullary carcinoma: a recently described highly aggressive renal tumor in young black patients. J Urol. 1997;157(6):2246-2247. doi:10.1016/s0022-5347(01)64734-7.
49. Friedrichs P, Lassen P, Canby E, Graham C. Renal medullary carcinoma and sickle cell trait. J Urol. 1997;157(4):1349.
50. Basher F, Dutcher G, England JS, Lopes G. Unusual presentation of renal medullary carcinoma with undiagnosed sickle cell trait. Cureus. 2020;12(9):e10731. doi:10.7759/cureus.10731.
51. Holland P, Merrimen J, Pringle C, Wood LA. Renal medullary carcinoma and its association with sickle cell trait: a case report and literature review. Curr Oncol. 2020;27(1):e53-e56. doi:10.3747/co.27.5043.
52. Chumdermpadetsuk R, Tooley AA, Godfrey KJ, et al. Renal medullary carcinoma with metastasis to the temporal fossa and orbit. Ophthalmic Plast Reconstr Surg. 2019;35(6):e149-e151. doi:10.1097/IOP.0000000000001478.
53. Toriyama A, Izumi H, Tomita S, et al. Renal medullary carcinoma in a young mixed-race man in Japan. Pathol Int. 2019;69(4):241-245. doi:10.1111/pin.12783.
54. Goenaga-Vázquez Y, Colón G, Barrios N, Correa M. Renal medullary carcinoma: a nearly fatal malignancy specifically affecting patients with a so-called benign condition. CEN Case Rep. 2018;7(1):121-126. doi:10.1007/s13730-018-0308-3.
55. Ibilibor C, Medway A, Nelius T. Renal medullary carcinoma with an ophthalmic metastasis. Urol Ann. 2017;9(2):184-187. doi:10.4103/0974-7796.204184.
56. Kalavar MR, Ali S, Safarpour D, Kunnakkat SD. Renal medullary carcinoma with an aggressive clinical course: a case report and review of the literature. Case Rep Oncol. 2017;10(1):1-7. doi:10.1159/000455007.
57. Tripathy K, Misra A, Dash K. Cytodiagnosis of renal medullary carcinoma: report of a case with immunocytochemistry. Cytopathology. 2015;26(5):328-330. doi:10.1111/cyt.12225.
58. Daher P, Bourgi A, Riachy E, et al. Renal medullary carcinoma in a white adolescent with sickle cell trait. J Pediatr Hematol Oncol. 2014;36(5):e285-e289. doi:10.1097/MPH.0000000000000118.
59. Anne M, Sammartino D, Chaudhary S, Bhuiya T, Mehrotra B. Renal medullary carcinoma masquerading as bilateral breast carcinoma. World J Oncol. 2013;4(3):169-172. doi:10.4021/wjon676w.
60. Nava VE, Mullin BR, Schuldenfrei JA. Renal medullary carcinoma metastatic to forehead. Am J Dermatopathol. 2011;33(7):757. doi:10.1097/DAD.0b013e3182106f2c.
61. Walsh AM, Fiveash JB, Reddy AT, Friedman GK. Response to radiation in renal medullary carcinoma. Rare Tumors. 2011;3(3):e32. doi:10.4081/rt.2011.e32.
62. Diao B, Paule B, Esquivel S, et al. Renal medullary carcinoma: remission with gemcitabine-cisplatin and review of therapeutic perspectives. Prog Urol. 2010;20(7):538-541. doi:10.1016/j.purol.2009.11.005.
63. Neville A, Hatem SF. Renal medullary carcinoma: unsuspected diagnosis at stone protocol CT. Emerg Radiol. 2007;14(4):245-247. doi:10.1007/s10140-007-0596-y.
64. Molgat G, Afrouzian M, Trpkov K. Renal medullary carcinoma as an incidental finding in a horseshoe kidney: case report and literature review. Can J Urol. 2005;12(5):2837-2840.
65. Johal NS, Desai D, Cuckow PM. Renal medullary carcinoma: beware of diagnosing a urinary tract infection in a young sickle cell patient. Hosp Med. 2005;66(2):114-115. doi:10.12968/hmed.2005.66.2.17561.
66. Zdinak LA, Nik NA, Hidayat AA, Hargett NA. Renal medullary carcinoma metastatic to the orbit: a clinicopathologic report. Ophthalmic Plast Reconstr Surg. 2004;20(4):322-325. doi:10.1097/01.iop.0000129530.75840.49.
67. Noguera-Irizarry WG, Hibshoosh H, Papadopoulos KP. Renal medullary carcinoma: case report and review of the literature. Am J Clin Oncol. 2003;26(5):489-492. doi:10.1097/01.coc.0000037663.61643.5A.
68. Selby DM, Simon C, Foley JP, Thompson IM, Baddour RT. Renal medullary carcinoma: can early diagnosis lead to long-term survival? J Urol. 2000;163(4):1238. doi:10.1016/s0022-5347(05)67733-6.
69. Pirich LM, Chou P, Walterhouse DO. Prolonged survival of a patient with sickle cell trait and metastatic renal medullary carcinoma. J Pediatr Hematol Oncol. 1999;21(1):67-69.
70. Adsay NV, deRoux SJ, Sakr W, Grignon D. Cancer as a marker of genetic medical disease: an unusual case of medullary carcinoma of the kidney. Am J Surg Pathol. 1998;22(2):260-264. doi:10.1097/00000478-199802000-00017.
71. Mathur SC, Schwartz AM. Pathologic quiz case: a 33-year-old man with an abdominal mass. Arch Pathol Lab Med. 2000;124(10):1561-1563. doi:10.5858/2000-124-1561-PQC.
72. Batra S. Is there a role for antiangiogenic therapy in renal medullary carcinoma? Pediatr Blood Cancer. 2016;63(4):746-747. doi:10.1002/pbc.25832.
73. Lipkin JS, Rizvi SM, Gatalica Z, et al. Therapeutic approach guided by genetic alteration: use of MTOR inhibitor in renal medullary carcinoma with loss of PTEN expression. Cancer Biol Ther. 2015;16(1):28-33. doi:10.4161/15384047.2014.972843.
74. Alappan N, Marak CP, Chopra A, et al. Renal medullary cancer in a patient with sickle cell trait. Case Rep Oncol Med. 2013;2013:129813. doi:10.1155/2013/129813.
75. Anazoeze M, Najibah G, Garba U, et al. Is renal medullary carcinoma the seventh nephropathy in sickle cell disease? A multi-center Nigerian survey. Afr Health Sci. 2016;16(2):490-496. doi:10.4314/ahs.v16i2.17.
76. Sathyamoorthy K, Teo A, Atallah M. Renal medullary carcinoma in a patient with sickle-cell disease. Nat Clin Pract Urol. 2006;3(5):279-283. doi:10.1038/ncpuro0483.
77. Dimashkieh H, Choe J, Mutema G. Renal medullary carcinoma: a report of 2 cases and review of the literature. Arch Pathol Lab Med. 2003;127(3):e135-e138. doi:10.5858/2003-127-e135-RMCARO.
78. Marsh A, Golden C, Hoppe C, Quirolo K, Vichinsky E. Renal medullary carcinoma in an adolescent with sickle cell anemia. Pediatr Blood Cancer. 2014;61(3):567. doi:10.1002/pbc.24795.
79. Sirohi D, Smith SC, Epstein JI, et al. Pericytic tumors of the kidney—a clinicopathologic analysis of 17 cases. Hum Pathol. 2017;64:106-117. doi:10.1016/j.humpath.2017.04.005.
80. Toriyama A, Izumi H, Tomita S, et al. Renal medullary carcinoma in a young mixed-race man in Japan. Pathol Int. 2019;69(4):241-245. doi:10.1111/pin.12783.
81. Gatalica Z, Lilleberg SL, Monzon FA, et al. Renal medullary carcinomas: histopathologic phenotype associated with diverse genotypes. Hum Pathol. 2011;42(12):1979-1988. doi:10.1016/j.humpath.2011.02.026.
82. Huang ZM, Wang H, Ji ZG. Renal medullary carcinoma masquerading as renal infection: a case report. BMC Nephrol. 2020;21(1):79. doi:10.1186/s12882-020-01730-y.
83. Al-Daghmin A, Gaashan M, Haddad H. Atypical presentation of renal medullary carcinoma: a case report and review of the literature. Urol Case Rep. 2018;22:8-10. doi:10.1016/j.eucr.2018.09.023.
84. Kalyanpur A, Schwartz DS, Fields JM, et al. Renal medulla carcinoma in a white adolescent. AJR Am J Roentgenol. 1997;169(4):1037-1038. doi:10.2214/ajr.169.4.9308460.
85. Çalışkan S, Gökçe AM, Gümrükçü G, Önenerk M. Renal medullary carcinoma: a rare entity. Iran J Med Sci. 2017;42(2):215-218.
86. Rodriguez-Jurado R, Gonzalez-Crussi F. Renal medullary carcinoma. J Urol Pathol. 1996;4:191-203.
87. Heng DY, Chi KN. Primary systemic therapy for synchronous metastatic renal cell carcinoma: the role for cytoreductive nephrectomy. J Oncol Pract. 2007;3(3):123-125.
88. Tsuzuki S, Kataoka TR, Ito H, et al. A case of renal cell carcinoma unclassified with medullary phenotype without detectable gene deletion. Pathol Int. 2019;69(12):710-714. doi:10.1111/pin.12858.
89. Di Stefano RF, Buttigliero C, De Luca E, et al. Metastatic renal medullary carcinoma treated with immune checkpoint inhibitor: case report and literature review. Clin Genitourin Cancer. 2018;16(6):e1087-e1090. doi:10.1016/j.clgc.2018.07.011.
90. Lai JZ, Lai HH, Cao D. Renal cell carcinoma, unclassified with medullary phenotype and synchronous renal clear cell carcinoma present in a patient with no sickle cell trait/disease: diagnostic and therapeutic challenges. Anticancer Res. 2018;38(6):3757-3761. doi:10.21873/anticanres.12657.
91. Mast Vilaseca R, Pons Escoda A. Carcinoma medular renal en un adulto joven, de raza blanca y sin enfermedad ni rasgo de células falciformes. Radiologia. 2015;57(4):361-363. doi:10.1016/j.rx.2014.09.003.
92. O'Donnell PH, Jensen A, Posadas EM, et al. Renal medullary-like carcinoma in an adult without sickle cell hemoglobinopathy. Nat Rev Urol. 2010;7(2):110-114. doi:10.1038/nrurol.2009.255.
93. Halilu F, Guru S, Fuscaldo J. The not-so-benign sickle cell trait: a case of renal medullary carcinoma. J Community Hosp Intern Med Perspect. 2024;14(3):60-64. doi:10.55729/2000-9666.1331.
94. Dalela D, Batie S, Carolan A, et al. Renal medullary carcinoma: the zebra amongst the horses. Urology. 2024;187:78-81. doi:10.1016/j.urology.2024.02.048.
95. Hou G, Jiang Y, Wang J, Li F, Cheng W. Renal medullary carcinoma on dual-time point FDG PET/CT imaging. Clin Nucl Med. 2020;45(6):446-447. doi:10.1097/RLU.0000000000003033.
96. Sarkar S, Throckmorton W, Bingham R, et al. Renal cell carcinoma unclassified with medullary phenotype in a patient with neurofibromatosis type 2. Curr Oncol. 2023;30(3):3355-3365. doi:10.3390/curroncol30030255.
97. Noreña-Rengifo BD, Ochoa-Gaviria J, Vélez-Escobar A, et al. Renal medullary carcinoma in an adolescent with unknown sickle cell trait. Cureus. 2021;13(4):e14473. doi:10.7759/cureus.14473.
98. Valeri M, Cieri M, Elefante GM, et al. Unclassified renal cell carcinoma with medullary phenotype and SMARCB1/INI1 deficiency: broadening the spectrum of medullary carcinoma. Front Med (Lausanne). 2022;9:835599. doi:10.3389/fmed.2022.835599.
99. Luthra M, Bayne F. The seventh sickle cell nephropathy. Intern Med. 2010;49:2641. doi:10.2169/internalmedicine.49.4479.
100. Vargas-Gonzalez R, Sotelo-Avila C, Coria AS. Renal medullary carcinoma in a six-year-old boy with sickle cell trait. Pathol Oncol Res. 2003;9(3):193-195. doi:10.1007/BF03033737.
101. Friedrichs P, Lassen P, Canby E, Graham C. Renal medullary carcinoma and sickle cell trait. J Urol. 1997;157(4):1349.
102. Courcier J, De La Taille A, Bertolo R, et al. Surgical and oncological management of renal medullary carcinoma in a young patient: a case report. Front Oncol. 2023;13:1073728. doi:10.3389/fonc.2023.1073728
103. Takeda M, Kashima S, Fuchigami Y, et al. Case report: a case of renal cell carcinoma unclassified with medullary phenotype exhibiting a favorable response to combined immune checkpoint blockade. Front Immunol. 2022;13:934991. doi:10.3389/fimmu.2022.934991.
